# Vulnerability of Older Adults in Natural Disaster Scenarios: A Case Study of Earthquakes

**DOI:** 10.1093/geroni/igaf122.097

**Published:** 2025-12-31

**Authors:** Wanwan Si, Hong Mi

**Affiliations:** Zhejiang University, Hangzhou, Zhejiang, China (People’s Republic); Zhejiang University, Hangzhou, Zhejiang, China (People’s Republic)

## Abstract

With the increasing frequency of extreme weather events and climate change, the impact of natural disasters on older adults has become a pressing social concern worldwide. Their vulnerability is not only associated with physiological decline but also influenced by social support systems, psychological resilience, and policy protections. Grounded in vulnerability theory, this study integrates perspectives from sociology, psychology, and medicine to examine the unique risks faced by older populations in disaster contexts. Using data from the EM-DAT International Disaster Database, the WHO Mortality Database, and the China Health and Retirement Longitudinal Study (CHARLS), this study selects three major earthquake events—the 2008 Wenchuan Earthquake in China, the 2011 Great East Japan Earthquake, and the 2016 Central Italy Earthquake—to systematically analyze how older adults experience disaster-related mortality, health impacts, social support availability, and governmental response measures across different countries. Findings highlight the role of diverse social security systems in shaping vulnerability among aging populations and explore strategies to reduce disaster risks, including optimizing emergency response policies, improving access to healthcare resources, and strengthening social support networks. This study provides theoretical insights and practical guidance for the development of age-inclusive disaster management policies, contributing to enhanced emergency preparedness, resilience, and post-disaster recovery for older adults.

